# The effects of electronic smoking on dental caries and proinflammatory markers: a systematic review and meta-analysis

**DOI:** 10.3389/froh.2025.1569806

**Published:** 2025-04-11

**Authors:** Abedelmalek Kalefh Tabnjh, Sara Alizadehgharib, Guglielmo Campus, Peter Lingström

**Affiliations:** ^1^ Department of Cariology, Institute of Odontology, Sahlgrenska Academy, University of Gothenburg, Gothenburg, Sweden; ^2^ Department of Applied Dental Sciences, Faculty of Applied Medical Sciences, Jordan University of Science and Technology, Irbid, Jordan; ^3^ Department of Oral Microbiology and Immunology, Institute of Odontology, Sahlgrenska Academy, University of Gothenburg, Gothenburg, Sweden

**Keywords:** dental caries, e-cigarettes, electronic smoking, proinflammatory markers, vaping

## Abstract

**Introduction:**

Smoking and the use of electronic cigarettes (e-cigs) are common practices that have significant consequences for oral health. Although the negative impact of traditional tobacco products on oral tissues is widely known, the emergence of e-cigs poses a new obstacle. This review summarises existing data on the influence of e-cigs on oral health, with a specific emphasis on dental caries and pro-inflammatory agents.

**Methods:**

A comprehensive search was conducted via PubMed, Web of Science, Embase, and Scopus to identify relevant studies published until September 2024. The structured search strategy uncovered 42 articles that were read in full text. The included articles consisted of clinical trials, observational studies, and laboratory investigations that examined the impact of e-cig aerosol on oral bacteria and pro-inflammatory markers and its potential to contribute to dental caries.

**Results:**

The findings indicate that e-cig users may have a higher prevalence of dental caries compared with non-smokers. Most studies focusing on bacteria showed that vaping may inhibit normal flora, giving cariogenic bacteria a chance to grow more. This finding indicates a notable oral health risk associated with vaping. Meta-analyses suggest no effect of using e-cigs on the levels of TNF-α, IL-1β, IL-6, and IL-8 in saliva, even if it may affect their levels in GCF. However, in GCF, only one study reported TNF-α and IL-1β, and only two studies reported IL-6 and IL-8. Nevertheless, the effects of e-cigs on dental caries require further investigation since the data do not provide a clear picture.

**Discussion:**

This review emphasises the necessity for ongoing research to clarify the mechanisms that cause these consequences and to guide public health policies aimed at reducing the harm caused by e-cigarettes.

**Systematic Review Registration:**

https://www.crd.york.ac.uk/PROSPERO/view/CRD42024537910, PROSPERO (CRD42024537910).

## 1 Introduction

Smoking is an epidemic habit that leads to behavioural, psychological, and physical dependence, similar to the use of other drugs such as alcohol, cocaine, and heroin (1). According to the Pan American Health Organization (PAHO) indicators for 2019, the tobacco epidemic claims the lives of over 8 million people annually, with more than 7 million deaths resulting directly from tobacco use and over 1.2 million from nonsmokers exposed to second-hand smoke (1). All forms of smoking have several well-documented negative impacts on the oral cavity (2), including dental caries, periodontal disorders, poor wound healing, and precancerous lesions that increase risk of oral carcinoma (3).

As an alternative to conventional tobacco products, tobacco-free electronic cigarettes (Electronic Nicotine Delivery Systems, ENDS), commonly referred to as e-cigarettes or vape pens, have gained popularity in recent years (4). The National Cancer Institute lists the components of an electronic cigarette as follows: a battery, a tank for holding liquid, a resistor for heating the liquid, a wick for absorbing liquid, and a nozzle used to inhale the produced aerosol. Among the components that may be included in the liquid within the storage tank are flavour additives (e.g., menthol, blueberry, and cinnamon), chemical additives (e.g., propylene and polyethylene glycol), and nicotine (1). Typically, organic cotton, used to absorb the liquid, is wrapped around a resistor—a high-temperature resistive wire alloy made of FeCrAl such as kanthal™. The battery provides the energy to heat the resistor, which creates the aerosol (5). A sensor detects airflow when the user inhales from the device, triggering the heating of the liquid in the cartridge, which leads to its evaporation. The vapor delivers nicotine to the user. Some of the nicotine can escape into the surrounding air when exhaled. The vapor temperature ranges between 40°C and 65°C. Manufacturers claim that a cartridge can provide ten to 250 puffs, equivalent to five to 30 cigarettes, depending on the brand (5).

There is growing concern about the potential effects of e-cigarettes on oral and dental health as their popularity has significantly increased in recent years (6). That is, there seems to be a connection between vaping and periodontal health (7). Studies on periodontal health have found that e-cigarette users, compared to non-tobacco users, exhibit higher levels of plaque index, clinical attachment loss, probing depth, and marginal bone loss (8). Additionally, a laboratory-based control study has demonstrated that oral fluids, such as gingival crevicular fluid (GCF) and unstimulated whole saliva, from nicotine users, express higher levels of immunoinflammatory biomarkers, including receptor activator of NF-kappa B ligand, interleukin (IL)-1β, and tumour necrosis factor-alpha (TNF-α) (8).

Research on the effects of vaping on dental caries remains limited. Following inhalation, some constituents of the vaping aerosol adhere to the soft and hard tissues of the oral cavity (9). Vaping generates a viscous aerosol that not only promotes biofilm formation and microbial adhesion but also alters the normal oral flora by inhibiting beneficial gram-positive bacteria and encouraging the growth of harmful gram-negative bacteria. The depletion of gram-positive bacteria in the oral cavity may contribute to caries development by facilitating the adhesion of *S. mutans* to the enamel (10).

Various e-liquids exhibit physical and chemical characteristics similar to sugary food (11). As sucrose is the main ingredient in many flavours, it can significantly increase both biofilm growth and enamel demineralization, findings confirmed by *in vitro* investigation (7). A major concern is that sucrose and other sugars are often not listed on product labels, potentially leading to consumer misunderstandings about the safety, risks, and contents of both unheated or heated e-cigarette liquids (12). Additionally, the glycerol and propylene glycol found in e-cigarettes promote water absorption, which may lead to xerostomia or dry mouth, further increasing the risk of dental caries (13).

In conclusion, many studies suggest that electronic cigarette use negatively affects oral health, including an increased risk of dental caries due to changes in normal flora, promotion of cariogenic bacteria, biofilm adhesion, sugar production, and xerostomia (7, 14–17). These findings highlight the need for further research to clarify the relationship between vaping and dental caries. This systematic review explores the effects of vaping on oral health, particularly on dental caries and proinflammatory markers.

## 2 Materials and methods

### 2.1 Study registration and format

The review methodology followed to PRISMA principles (18) and was registered in PROSPERO (CRD42024537910) (19).

### 2.2 Population, exposure, comparison and outcomes (PECOs)

The following PECOs guided the formulation of the research question: P (population)—human cells or oral bacteria exposed to e-cigs or smokers who use e-cigs; E (exposure)—smoking electronic cigarettes or the vapour produced by them; C (comparison)—smokers (CS) and non-smokers (NS); and Os (outcomes)—both clinically evaluated and user-reported changes in proinflammatory markers and variables associated with dental caries caused by e-cigs.

### 2.3 Inclusion and exclusion criteria

The following inclusion criteria were used: observational and interventional studies on e-cigs and its effects on dental caries or proinflammatory markers; studies including any age or sex; laboratory studies considering oral bacteria or human oral cells; and studies written in English, without time limits.

The following exclusion criteria were used: all review studies and meta-analyses; case reports or case series studies; studies with unclear information; papers not focusing on dental caries or proinflammatory markers; comment articles; and posters or conference abstracts.

To prevent unit-of-analysis mistakes in cases where some of the studies included data from paired or repeated observations of participants, The Cochrane Handbook for Systematic Reviews of Interventions (Section 9.3.3) was consulted (20).

### 2.4 Search strategies

For every database, a specific search plan was created, considering variations in syntax rules and restricted vocabulary (AT). The search strategy used for each database is provided in the Supplementary Material.

### 2.5 Electronic search

One author (AT) conducted the electronic search across four databases: PubMed, Embase, Web of Science, and Scopus. The search was performed in April 2024 and updated in September 2024. Endnote 21® software was used to check all references for duplicates and study selection. This resulted in more relevant records, which were manually searched using the reference lists of the listed studies.

### 2.6 Study selection

After removing duplicates, two authors (AT and SA) independently screened the references by title and abstract using the Systematic Review Accelerator website (https://pubmed.ncbi.nlm.nih.gov/32004673/) (21). In case of a disagreement, confirmation was obtained after consulting with a third author (PL). Cohen's Kappa value was used to determine the agreement between the two screeners.

### 2.7 Data extraction

The extracted data summarised the studies into three major categories: studies focusing on caries-related variables (Table 1), studies focusing on cariogenic bacteria (Table 2), and studies focusing on proinflammatory markers (Table 3). Table 1 lists the variables: age, study type, records type (clinical or surveys), the country of the study, groups included in the study, sample size, study parameters, and outcomes. Table 2 lists the variables: country, study design and type, study groups, study aims, and conclusion. Table 3 lists the variables: age, sex, study design, inclusion criteria for the ES group, the study groups, sample size, the aim of the study, the study parameters, and the conclusion.

**Table 1:** Studies on vaping and dental caries-related variables from PubMed, Embase, Web of Science, and Scopus, up to September 2024, *N* = 16.

#	Author	Year	Study design	Study type	Study group	Country	Age group (mean age)	Total number	ES group	Study parameters	Outcomes
1	Ghazaly et al. (22)	2019	Observational (6 m)	Clinical	ES, CS, NS	Malaysia	Adults (22.9)	135	45	DMFT	DMFT = ES: 3.13, CS: 4.09, NS: 3.51.
2	Ghazaly et al. (3)	2018	Observational	Clinical	ES, CS, NS	Malaysia	Adults (*m* = 22.9)	120	40	DMFT, GI, PI, BOD, CAL	DMFT = S: 3.05 (1.66), CS: 3.23 (3.92), NS: 3.65 (3.76).
3	Irusa et al. (7)	2022	Cross-sectional	Data Records	ES, Non ES	USA	>16	13,098	91	CAMBRA	CAMBRA = ES (Low: 6.6%, Mod: 25.8%, High: 79.1%); Non-ES (Low: 14.5%, Mod: 25.9%, High: 59.6%; OR: 0.36 [ES as Ref.]).
4	Vemulapalli et al. (17)	2021	Cross-sectional	Data Records	ES, CS, DS, FS, NS	USA	≥18	4,618	ES = 24, DS = 120	Untreated caries	Untreated Caries OR for ES = 2.04 [NS as Ref.]
5	Fairchild & Setarehnejad (12)	2021	Experimental	Vitro	E-cig fluids	UK	NA	45	NA	pH	pH < 5.5 = 38 (84%).
6	Chaffee et al. (23)	2021	Observational (12 m)	Questionnaire	ES, CS, Cannabis	USA	Adolescents	964	116	Dry mouth	Dry mouth = ES (12%, OR: 1.4) (Ref. is no dry mouth).
7	Fagan et al. (13)	2017	Analytical	Vitro	E-cig fluids	USA	NA	66	NA	Sugars & aldehyde content	Sucrose *m*(sd) = 125 (153.7) ug/ml, Glucose = 20.4 (20.4)) ug/ml, Fructose = 61.3 (79.9) ug/ml.
8	Kubica et al. (24)	2014	Analytical	Vitro	E-cig fluids	Poland	NA	37	NA	Sucrose	Sucrose Range = (0.76–72.93) ug/g.
9	Cichonska et al. (25)	2022	Cross-sectional	Clinical	ES, CS, NS	Poland	20–30	128	40	pH, Total protein, Calcium, Phosphate (in saliva)	ES group have a significate higher Calcium compared to CS and NS, while no significant diff. found for pH, protein, and phosphate.
10	Palomino et al. (26)	2017	Experimental	Vitro	E-cig fluids	Brazil	NA	63	Diff. flavours	Enamel colour change	Delta-E *m*(sd) = Neutral: 2.4 (1.1), Menthal: 4.6 (1.8), Tobacco Flv: 3.1 (1.3).
11	Alamer et al. (14)	2024	Cross-sectional	Data Records	CS, ES, Cigars, NS	USA	≥30	7,840	119	Untreated Caries	Untreated Caries = ES: (Cornal: 32.61% OR: 4.21, Root: 25.59% OR: 2.48), CS: (Coronal: 33.91% OR: 3.78, Root: 27.9% OR: 2.84), NS: (Cornal: 12.14% OR: 1 Ref, Root: 7.49%).
12	Alhaj et al. (27)	2022	Cross-sectional	Questionnaire	EC, CS, DS, NS	Yemen	Around 20 (dental students)	5,697	261	DMFT, Dry mouth	DMFT for ES = Non: 77 (30.2%), <3: 92 (36.2), ≥3: 86 (33.7), Dry mouth = ES: 33.3%, Control: 23.4%.
13	Ko & Kim (28)	2022	Experimental	Vitro	E-cig with diff temp (flavourless)	USA	NA	NA	NA	pH, Viscosity, Colour, Metals	Vaping temp. Affect: Aerosols viscosity (Adhesion), Colour, Metal, but not pH.
14	Zhao et al. (21)	2019	Experimental	Vitro						Delta E for Enamel & Dentin	Delta-E *m*(sd) = Enamel: −2.27 (0.53), Dentin: −2.81 (0.91).
15	Alyaseen & Aldhaher (29)	2024	Observational	Clinical	ES, NS	Iraq	18–25	90	45	DMFS	DMFS = ES: 15.53 (2.75), NS: 6.96 (2.91).
16	Zieba et al. (30)	2024	Cross-sectional	Clinical	EC, CS, HTS, NS	China	18–36	113	27	DMFT	DMFT = ES: 17 (0.23) (1.66), CS: 18 (0.32), HTS: 18 (0.28), NS: 17 (0.31).

ES, electronic smokers; DS, dual smokers; CS, cigarette smokers; NS, non-smokers; FS, former smokers; HTS, heated tobacco smokers; DMFT/S, decayed, missing, filling, teeth/surfaces; BOP, bleeding on probing; GI, gingival index; CAL, clinical attachment loss; PI, plaque index; CAMBRA, caries management by risk assessment; OR, odd ratio; NA, not applicable.

**Table 2:** Studies on vaping and bacteria from PubMed, Embase, Web of Science, and Scopus, up to September 2024, *N* = 9.

#	Author	Year	Country	Study design	Study type	Study group	ES groups	Study aim	Conclusion
1	Fischman et al. (31)	2020	USA	Experimental	Vitro	E-cig fluids	Different Flavors	E-liquids and FEC aerosols may alter commensal oral *streptococcal* bacteria growth compared to unflavoured.	FEC affect oral commensal bacteria more than UFEC.
2	Valentin et al. (32)	2022	USA	Experimental	Vitro	E-cig fluids	Non-Nic, Nic, FEC, UFEC	If E-Cigs Promote Oral S. mutans Over Commensal Streptococci.	E-cig aerosols may disrupt oral bacterial balance by inhabiting commensal growth and *S. mutans* biofilm formation.
3	Xu C. et al. (33)	2022	USA	Experimental	Vitro	E-cig fluids	Different Flavors	Biofilm development and toxicity of four oral commensal bacteria species: *S. gordonii*, *S. intermedius*, *S. mitis*, and *S. oralis*.	Elevated concentrations FEC inhibits biofilm formation and the spread of oral commensal streptococci, both in single and multi-species communities.
4	Kim et al. (15)	2018	USA	Experimental	Vitro	E-cig fluids	Different Flavors	To examine teeth cariogenic potential after FEC aerosol exposure.	E-liquid viscosity and sweet flavour compounds may enhance cariogenic risk.
5	Nelson et al. (34)	2019	USA	Experimental	Vitro	CS, ES (Nic/Non-Nic), Liquid Nic	Nic & Non-Nic	The study compares the effects of UFEC aerosol on oral commensal streptococci development to those of CS.	CS is more harmful to oral commensal streptococci growth and biofilm formation than UFEC aerosol or liquid nicotine.
6	Rouabhia & Semlali (16)	2021	Canada	Experimental	Vitro	E-cig (Nic/Non-Nic)	Nic & Non-Nic	To determine how E-cigs affect *S. mutans* growth, biofilm formation, and virulence gene expression.	E-cigs enhanced *S. mutans* growth and pathogenic gene expression. Biofilms on dental surfaces were promoted by e-cigs.
7	Cuadra et al. (35)	2019	USA	Interventional	Vitro	UFEC (N/NN), CS	UFEC (Nic/Non-Nic)	To compare UFEC aerosol and CS smoke on oral commensal streptococci survival and growth.	Unflavoured has small effect
8	Tishchenko et al. (36)	2022	Ukraine	Cross-sectional	Clinical	EC, CS, NS	20	To determine dental microbiocenocis shifts in ENDS-using adolescents.	ENDS increase excretion frequency and opportunistic transitory streptococci, reducing plaque microflora.
9	Liu et al. (37)	2024	China	Experimental	Vitro	EC, CS, NS	27	Investigating the effects of using CS and E-cig on oral flora	CS and E-cig changed the structure and composition of the oral microbiome.

E-cigs, electronic cigarettes; ES, electronic smokers; DS, dual smokers; NS, non-smokers; CS, cigarettes smokers; WS, waterpipe smokers; FS, former smokers; FEC, flavoured electronic cigarettes; UFEC, unflavoured electronic cigarettes; Nic, nicotine.

**Table 3:** Studies on vaping and proinflammatory markers from PubMed, Embase, Web of Science, and Scopus, up to September 2024, *N* = 17.

#	Author	Year	Country	Study design	Criteria for ES group	Study group	Age group	Male%	Total number	ES group	Aim	Study parameters	Conclusion
1	BinShabaib et al. (38)	2019	Saudi	Observational (Case-Control)	Subjects who only use electronic cigarettes at least once a day.	ES, CS, Control	Adults (*m* = 42.2)	95.5	135	44	Comparison of clinical periodontal condition and GCF cytokine profile.	PI, PD, BOP, CAL, MBL, IL-1β, IL-6, TNF-α, IFN-γ, MMP-8.	Periodontal status is poorer and GCF levels of proinflammatory cytokines are higher in CS than EC and NS. However, vaping is not a healthy substitute for smoking.
2	Wadia et al. (39)	2016	UK	Interventional	Healthy, Smoked at least 10 cig/d.	ES	18–65	NA	20	18	To compare the gingival health of established smokers before and after switching to ES.	PD, BOP, PI, IL-1β, IL-8, IL-6 (GCF, Saliva and blood)	ES significantly increase BOP
3	Mokeem et al. (40)	2018	Saudi	Cross-Sectional	Self-reported smoking status.	ES, CS, WS, NS	Adults (*m* = 28.3)	100	154	37	To compare periodontal and inflammatory marker changes.	PI, PD, BOP, CAL, MBL, IL-1β, IL-6, cotinine	Parameters of periodontal inflammation were poorer in CS and WS than ES and NS.
4	Ye et al. (41)	2020	USA	Cross-Sectional	Excluded: Patients with inflammatory diseases, individuals requiring antibiotic prophylaxis for routine dental procedures, and those who have received antibiotics within the past 3 months.	ES, CS, DS, NS	Adults (*m* = 34.9)	83.3	48	12	To compare inflammatory markers in saliva and GC.	IL-1β, PGE2, cotinine, All GCF biomarkers	Smoking/vaping produces differential effects on oral health.
5	Thomas et al. (42)	2022	USA	Observational (6 m)	At least mild periodontitis and did not receive prophylactic cleaning during the study period	ES, CS, NS	Adults (*m* = 37)	78.6	84	28	Assessing the periodontal risks of E-cig use.	Bacteria, IL-(1β, a, 2, 4, 6, 8, 10, 12p70, and 13), TNF-α, IFN-γ, BOP, PD, Cotinine	General correlate with cytokine and clinical measures
6	Karaaslan et al. (43)	2020	Turkey	Cross-Sectional	(i) Individuals with periodontitis; (ii) ES: Participants were ex-smokers who smoked more than 10 T-cigs/day for 10 years and vaped E-cigs for 12 months.	ES, CS, FS	Adults (*m* = 34.7)	68.4	57	19	To evaluate how ES, CS, and smoking cessation affect oxidative stress, proinflammatory cytokines, and periodontitis markers.	PI, PD, GI, CAL, IL-8, GSH-Px, TNF-α, 8-OHdG	ES and CS both negatively affected oxidative stress and inflammatory cytokines.
7	Faridoun et al. (44)	2020	USA	Cross-Sectional	None	ES, CS, DS, NS	28–83	57.8	64	15	To compare inflammatory biomarkers in saliva between groups	IL-(6, 8, 10, 1β, 1RA), TNF-α, C-reactive protein	The findings put into question the safety of e-cigs as a smoking cessation mechanism
8	Ganesan et al. (45)	2020	USA	Cross-Sectional	Who used E-cigs daily for at least 3 months, with at least 1cartridge/day or 1 ml/day.	ES, CS, DS, FS-ES, NS	NA	NA	123	20	To study how ES affects the subgingival microbiota and the host's immunoinflammatory response.	IL-(2, 4, 6, 8, 10), IFN-γ, GM-CSF, TNF-α	The study raises concerns about the safety of ES and the idea that it reduces harm that is pushed in advertising.
9	Ali et al. (8)	2022	Kuwait	Observational (Case-Control)	individuals who had used ENDS at least once in the past 30 days	ES, CS, NS-PD, NS	Adults (*m* = 49.5)	63.2	76	18	Comparing periodontal health and salivary IL-15 and -18 levels between groups.	IL-(15, 18), UWSFR, CAL, PI, PD, GI, BOP, MBL, MT	Salivary inflammatory indicators (IL-15 and -18) are higher in CS than NS, despite equivalent periodontal states.
10	Pushalkar et al. (46)	2020	USA	Cross-Sectional	Systemically healthy, an E-cig user (no smoking, 0.5–1 ml e-cig/day for 6 months).	ES, CS, NS	≥21	77	120	40	Compare risk of infection between groups.	Cotinine, bacteria, IL-(2, 4, 6, 8, 10, 13, 12p70, 1β), IFN-γ, TNF-α	ES users are more prone to infection.
11	Xu F. et al. (47)	2022	USA	Prospective (6 m)	E-cig users (never smoked and using 0.5–1 e-cig/day for past 6 months), have a minimum of 16 teeth, including 8 post-teeth, and diagnosed with mild, mod, or severe perio disease, and systemically healthy.	ES, CS, NS	≥21 (*m* = 37)	NA	101	40	To investigate the effect of ES use on the bacterial community structure in the saliva.	IL-(2, 4, 6, 8, 10, 12p70, 13, 1β), TNF-α, IFN-γ	ES may gradually alter the microbiota in periodontal disease patients like CS.
12	Alhumaidan et al. (48)	2022	Saudi	CRT	Solely using ENDS for the past 12 months, and healthy.	ES, CS, NS	Adults (*m* = 41.3)	66.7	54	18	Examine salivary CL and IL-1β levels in light CS and ES patients before and after NSPT.	PI, GI, PD, MT, CAL, MBL, Salivary Flow Rate, IL-1β, cortisol	Light CS and ES users without periodontal disease show no change in clinical periodontal markers, whole-salivary CL, or Il-1β levels following NSPT.
13	Ibraheem et al. (49)	2020	Saudi	Observational (Case-Control)	Vaping at least once daily for the past 12 months, and healthy.	ES, CS, WS, NS	Adults (*m* = 45.6)	100	120	30	To compare the levels of OPG and RANKL in GCF.	PI, BOP, CAL, PD, MBL, GCF (RANKL& OPG)	CS, WS, and ES promote GCF RANKL and OPG expression.
14	AlQahtani et al. (50)	2020	Saudi	Cross-Sectional	Healthy 18–40-year-olds denied recent dental surgery or oral disease.	ES, NS	18–40 (*m* = 20.36)	64.3	30	14	Compere salivary metabolites and proinflammatory.	IL-(6, 8, 1β), TNF-α, Saliva metabolites, cotinine	There are a differences in components of saliva including inflammatory cytokines and metabolites in ES compared to NS.
15	Kamal et al. (51)	2022	Egypt	Cross-Sectional	ES exclusively for at least 12 months and had never CS before.	ES, CS, NS	Adults (*m* = 29.36)	64	150	50	To investigate the effects of ES on the salivary biomarkers.	IL-1β, TGF-B	Type of smoker can influence some of the detectable inflammatory biomarkers.
16	Alkhalifah et al. (52)	2024	Kuwait	Cross-Sectional	Gingivitise, at least 20 teeth.	ES, NS	18–25	100	38	20	To compare the effect of ultrasonic scaling on the expression of IL-1β in the GCF.	IL-1β, PD, BOP, PI	ENDS increase excretion frequency and opportunistic transitory streptococci, reducing plaque microflora.
17	Zieba et al. (30)	2024	Poland	Cross-Sectional	1–3 years smoking, Healthy, Normal BMI	ES, CS, HTS, NS	<30 (*m* = 24.5)	NA	100	25	To assess how CS, EC, and HS affect salivary cytokines, chemokines, and growth factors in healthy young people.	PD, TNF-α, IFN-γ, IL-1β, IL-6, IL-8	CS and EC changed the structure and composition of the oral microbiome.

E-cigs, electronic cigarettes; ES, electronic smokers; NS, non-smokers; WS, waterpipe smokers; DS, dual smokers; CS, cigarette smokers; FS, former smokers; HTS, heated tobacco smokers; PD, probing pocket depth; BOP, bleeding on probing; PI, plaque index; GI, gingival index; CAL, clinical attachment loss; IL, inter leucine; GCF, gingival crevicular fluid; NA, not available; NSPT, non-surgical periodontal treatment; MD, mechanical debridement; BI, bleeding index; MT, missing teeth; MBL, marginal bone loss; IFN-γ, interferon-gamma; MMP-8, matrix metalloproteinase; TNF-a, tumour necrosis factor-a; PGE2, prostaglandin E2; 8-OHdG, 8-hydroxydeoxyguanosine; GSH-Px, glutathione peroxidase; GM-CSF, granulocyte-macrophage colony-stimulating factor; RANKL, receptor activator for nuclear factor *κ* B ligand; OPG, osteoprotegerin.

When the data were reported just as graphs, the *WebPlotDigitizer* tool was used (53). Moreover, data reported in forms other than means and SD (*e.g.*, median, IQR, standard error, or confidence intervals) were converted to mean and SD.

### 2.8 Risk of bias

**Figure 1:**
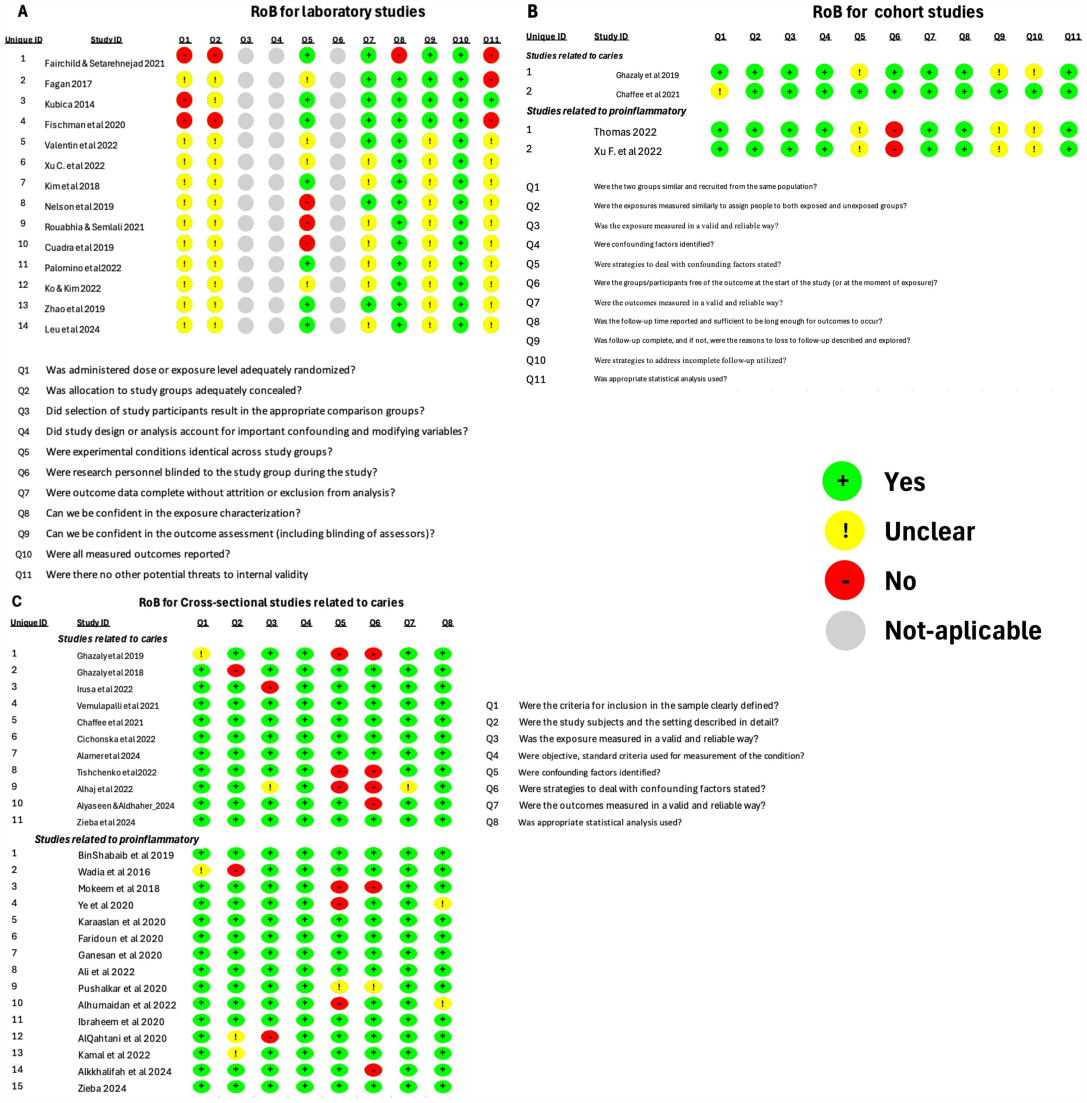
Risk of bias (RoB) analysis. **(A)** RoB for laboratory studies. **(B)** RoB for cohort studies. **(C)** RoB for cross-sectional studies.

The quality of cross-sectional and cohort studies was assessed using the JBI Critical appraisal tool (54). The Risk of Bias Approach for Laboratory Studies (55) was used for articles with an exclusive *ex vivo* or *in vitro* design. Two reviewers (AT and SA) conducted the assessments, and discussion resolved the differences in evaluations (Figure 1).

### 2.9 Data synthesis

Meta-analyses were carried out when at least three studies compared and reported the same data. Cohen's *D* effect size and 95% CIs were the main impact measures (56).

Analyses were done with SPSS 29.0.1.0. The random-effects model estimate of variance was adopted since considerable heterogeneity was expected between studies, and wider confidence intervals help deal with the influence of size uncertainty. The *I*^2^ statistics show the percentage of study variation caused by heterogeneity rather than chance. Heterogeneity was evaluated as low (<40%), moderate (40%–60%), substantial (61%–90%), and considerable (91%–100%) (20). To examine heterogeneity, we compared study variables such as participant, intervention, and result similarities as described in the inclusion criteria. Subgroup analysis was performed to explore the influence of study characteristics such as proinflammatory markers in different media (GCF and saliva).

## 3 Results

### 3.1 Search

**Figure 2:**
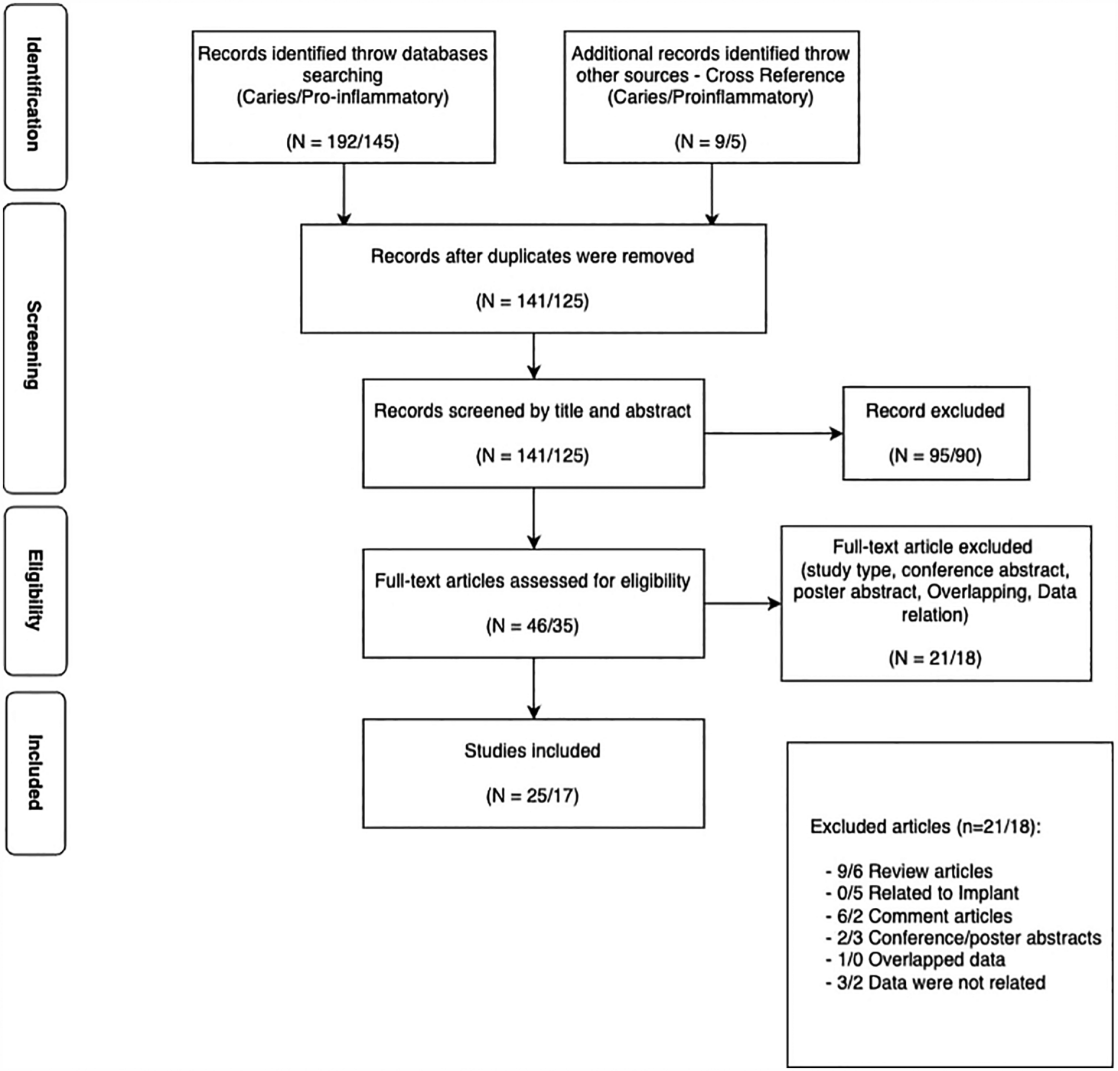
Flow diagram of the selection process of the studies included in the systematic review.

A total of 192 papers were retrieved for studies related to vaping and dental caries. With a manual search, nine more articles were discovered. After duplicates (*n* = 60) were removed, 141 articles were checked for title and abstract. The Cohen's Kappa value was 0.747 and the two authors' percentage of agreement was 90.8%. Finally, 46 articles were obtained in full-text, and 21 of these articles were eliminated after reading the full text because they did not meet the inclusion criteria. At the end, *N* = 25 (Figure 2). All of the studies reviewed were published between 2014 and 2024.

A total of 145 articles about vaping and proinflammatory-related studies were retrieved. Five additional articles were identified through cross-referencing. Following the removal of duplicates (*n* = 25), a total of 125 papers were screened based on their titles and abstracts. The inter-rater agreement between the two screeners was 96.6%, reflected by a Cohen's Kappa score of 0.909. A total of 35 papers were acquired in full-text format, and 18 of these were excluded following full-text reading due to non-compliance with the inclusion criteria. At the end, *N* = 17 (Figure 1). All included studies were published between 2018 and 2024. Every effort was made to acquire original data from the authors as required.

### 3.2 Data synthesis

Of the 25 studies focusing on caries, 14 studies were of laboratory design, nine were cross-sectional design, and two were cohort design. Sixteen studies focused on caries-related variables such as decayed, missing, and filled teeth (DMFT) index, sugar concentrations, dry mouth, untreated caries, pH, caries risk assessment, metal concentrations, and colour change. Five studies were based on questionnaires or data records, six were *in vitro* studies, and five were clinical studies (Table 1). The remaining nine studies focused on the effects of ENDS on cariogenic bacteria (Table 2). The main variables in those studies were bacterial growth, hardness loss, adhesion, and biofilm. *Streptococcus* species were the common types within all those articles.

Most of the 17 studies focusing on inflammation were cross-sectional (just two were cohort studies), comparing vaping groups with other groups such as tobacco smokers and non-smokers, and just three studies included the water-pipe smokers as a group. Of these, 12 studies measured the outcome in the saliva, four used GCF, and one, a pilot study, used saliva, GCF, and serum simultaneously (Table 3). The following were the most common proinflammatory markers and other measured parameters: volume, IL-1β, IL-6, IL-8, IFN-γ, TNF-α, UWSFR, and GCF volume. Other inflammatory markers included MMP-8, MMP-9, PGE2, TGF-β, OPG, GM-CSF, GsH-Px, 8-OHdG-IL, 8-OHdG-a, 8-OHdG-1RA, 8-OHdG-2, 8-OHdG-4, 8-OHdG-10, 8-OHdG-12p70, 8-OHdG-13, 8-OHdG-15, and 8-OHdG-18, and cotinine).

Most Laboratory and cohort studies showed an overall risk of bias that is probably moderate (12, 13, 15, 16, 21–24, 26, 28, 31–35, 37, 42, 47). On the other hand, all cross-sectional studies showed a “probably low” risk of bias (3, 7, 8, 14, 17, 22, 23, 25, 29, 30, 36, 38–41, 43–46, 48–52), with the exception of one study, which showed a “probably moderate” risk of bias (27).

### 3.3 Results of included studies

#### 3.3.1 Vaping and dental caries

Studies done regarding caries showed that users of ENDS (Electronic Nicotine Delivery Systems) may be at a higher risk for dental caries (7) and tend to have more untreated caries scores than non-smokers (14, 17). The results also indicate that users of ENDS experience dry mouth more frequently than non-smokers (23, 27). Studies that focused on ENDS's effects on bacteria revealed that ENDS inhibit normal flora without affecting *S. mutans* at concentrations of 5% or more, and this inhibition allows *S. mutans* to proliferate, increasing the risk of dental caries. However, ENDS with lower concentrations have minimal or no effect on oral *Streptococcus* species in the mouth (15, 16, 31–36).

#### 3.3.2 Vaping and proinflammatory factors

The results indicate a complex relationship between conventional smoking (CS), electronic smoking (ES), and proinflammatory factors. Compared to electronic cigarette smokers (ES) and non-smokers (NS), cigarette smokers (CS) generally exhibit worse periodontal conditions and higher levels of proinflammatory cytokines in GCF (39, 41). Both CS and ES have comparable detrimental effects on oxidative stress markers and inflammatory cytokines, and they are linked to increased expression of inflammatory biomarkers such as RANKL, OPG, IL-15, and IL-18 (8). The notion that ES is a safer smoking cessation aid is challenged by findings that people who use ES devices may also be more susceptible to infections and experience alterations in their oral microbiome, which could lead to oral imbalance (45, 46, 51, 52). However, when it comes to salivary proinflammatory markers such as IL-1β, IL-6, IL-8, and TNF-α, most studies reported no significant difference between users of ES devices and non-smokers (8, 40–42, 46, 51). These findings raise serious concerns about the safety of e-cigs and question the common harm reduction narrative associated with them, as the findings suggest that e-cigs negatively affect oral health in general and inflammatory responses in particular.

### 3.4 Meta-analysis

Meta-analyses were conducted for DMFT/DMFS (Decayed, Missing, Filled Teeth/Surfaces) scores and the most common proinflammatory markers, provided that at least three studies reported on each marker. The selected markers were IL-1β, IL-6, IL-8, and TNF-α. Cohen's *d* effect size was used to test the data. The meta-analyses also considered different media, including GCF and saliva, as subgroups.

#### 3.4.1 DMFT

**Figure 3:**
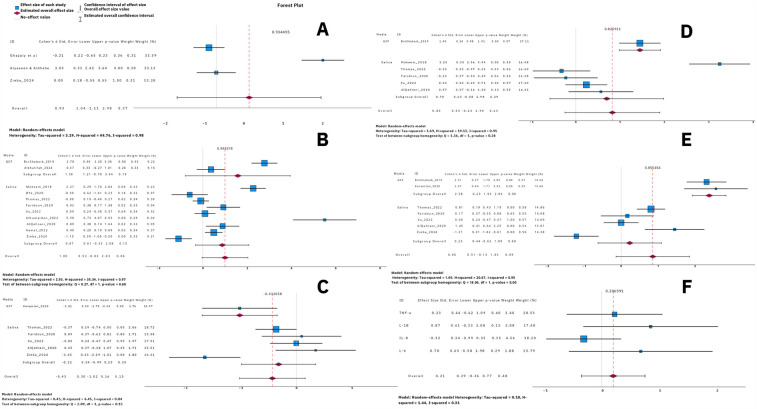
Forest plots for caries and proinflammatory markers. **(A)** DMFT, **(B)** IL-1β, **(C)** IL-8, **(D)** IL-6, **(E)** TNF-a, and **(F)** all salivary proinflammatory markers—IL-1β, IL-6, IL-8, and TNF-α.

A meta-analysis was conducted on three studies (22, 29, 30) that reported DMFT/DMFS (Figure 3A). A high heterogeneity was found between studies (*T*^2^ = 3.19, *I*^2^ = 0.98). Two studies reported DMFT (22, 30) and the other one reported DMFS (29).

#### 3.4.2 IL-1β

A meta-analysis was conducted on eleven studies (30, 38, 40–42, 44, 47, 48, 50–52) that reported IL-1β (Figure 3B). A high heterogeneity was found between studies (*T*^2^ = 2.93, *I*^2^ = 0.97). Two studies reported IL-1β in GCF and nine in saliva. The heterogeneity between saliva studies was high (*T*^2^ = 3.25, *I*^2^ = 0.97).

#### 3.4.3 IL-8

A meta-analysis was conducted on six studies (30, 42–44, 47, 50) that reported IL-8 (Figure 3C). A moderate heterogeneity was found between studies (*T*^2^ = 0.45, *I*^2^ = 0.84). One study reported IL-8 in GCF and five in saliva. The heterogeneity between saliva studies was moderate (*T*^2^ = 0.49, *I*^2^ = 0.86).

#### 3.4.4 IL-6

A meta-analysis was conducted on six studies (38, 40, 42, 44, 47, 50) that reported IL-6 (Figure 3D). A high heterogeneity was found between studies (*T*^2^ = 1.69, *I*^2^ = 0.95). One study reported IL-6 in GCF and five in saliva. The heterogeneity between saliva studies was high (*T*^2^ = 2.03, *I*^2^ = 0.95).

#### 3.4.5 TNF-α

A meta-analysis was conducted on seven studies (30, 38, 42–44, 47, 50) that reported TNF-α (Figure 3E). A high heterogeneity was found between studies (*T*^2^ = 1.96, *I*^2^ = 0.95). Two studies reported TNF-α in GCF and five in saliva. The heterogeneity between saliva studies was high (*T*^2^ = 0.86, *I*^2^ = 0.91).

#### 3.4.6 For all markers

A meta-analysis was conducted on all the proinflammatory markers (IL-1β, IL-6, IL-8, and TNF-α) in saliva using the effect size from the meta-analysis for each marker (Figure 3F). Low heterogeneity was found between the markers (*T*^2^ = 0.1, *I*^2^ = 0.31). The results suggest that vaping does not affect the level of these markers in saliva.

## 4 Discussion

This systematic review explores the effect of vaping on oral health in general, with a particular focus on dental caries and proinflammatory markers. The findings indicate that the effect of ENDS on dental caries and proinflammatory markers remains unclear and therefore requires further investigation. Most studies included in the meta-analysis had the same risk of bias and shared similar designs, so there was no need for sensitivity analysis.

The review identified various studies that examined multiple criteria related to dental caries. The impact of vaping on the increased risk of dental caries is a concerning trend. Some studies have demonstrated that users of ENDS exhibit a greater prevalence of dental caries compared to non-smokers (29). Furthermore, users ENDS reported a higher prevalence of untreated caries and xerostomia, potentially elevating the risk of dental caries (14, 17, 23, 27). Numerous studies have suggested that vaping alters the composition of the oral microbiota (15, 16, 31–33, 36, 37). These studies indicate that vaping may disrupt the normal flora, facilitating the growth of cariogenic bacteria such as *S. mutans*, which may replace healthy flora. The change in microbial composition and increased biofilm adherence and sugar production create an environment conducive to tooth demineralization and the development of dental caries. Furthermore, the findings indicate that the concentration of vaping liquid and flavours may influence the degree of caries risk (31, 33, 35). Higher concentrations appear to cause greater alterations in the normal flora. However, the evidence remains ambiguous, and further research is needed to better understand how vaping increases the risk of dental caries and to establish causative links.

Some studies suggest that vaping is associated with changes in some proinflammatory markers (41–46), but some studies report conflicting findings (38–40, 44, 47, 48, 51). A meta-analysis of common markers reported in this study (IL-1β, IL-6, IL-8, and TNF-α) found no significant association between vaping and these markers in saliva. However, potential connections were observed in GCF. After reviewing the outlier studies, we found that most of the heterogeneity stemmed from studies with small standard deviations, which had a significant effect size despite means similar to other studies.

The implications of these findings are significant for global health and the dental practice. As vaping continues to gain popularity, dental professionals should be aware of its potential oral health consequences. This study suggests that while ENDS may be less harmful in certain respects compared to conventional smoking, ENDS are not a completely safe alternative as they may be associated with negative oral health effects. Consequently, comprehensive education on the potential risks of vaping is crucial, particularly for young people and teenagers.

### 4.1 Limitations

Despite the significance of the findings of this study, several limitations must be considered. The heterogeneity in study design, participants, and methodologies complicates drawing definitive conclusions. Furthermore, several studies failed to adequately account for confounding factors, which compromises the validity of the results. Moreover, the reliance on self-reported data in several studies raises concerns about accuracy.

Future research should use a longitudinal design to focus on the long-term impacts of ENDS on oral health. Investigating how vaping impacts normal flora and inflammatory responses will elucidate the pathways contributing to dental caries. Furthermore, future studies should explore the effects of various vaping devices, temperatures, and flavours, as these elements may significantly influence oral health. In addition, research should aim to standardise methods and include larger, more diverse populations to improve generalizability.

## 5 Conclusion

The conclusions from this study highlight the possible adverse effects of ENDS on oral health, particularly concerning proinflammatory indicators and dental caries. The findings suggest that vaping may increase the risk of dental caries due to alterations in the normal oral flora and the growth of cariogenic bacteria. Although the meta-analyses indicated no significant impact of vaping on major salivary proinflammatory markers, they suggested a potential effect on salivary markers in the GCF. However, research on GCF remains limited. The existing literature is inconsistent and limited, indicating the need for further studies to better understand these associations and inform public health strategies aimed at reducing the risks associated with vaping.

## Data Availability

The original contributions presented in the study are included in the article/Supplementary Material, further inquiries can be directed to the corresponding author.
