# Supplementary material for: The effects of electronic smoking on dental caries and proinflammatory markers: a systematic review and meta-analysis
Source: Front Oral Health. 2025 Apr 11;6:1569806. doi: 10.3389/froh.2025.1569806 (PMC12021849; doi:10.3389/froh.2025.1569806)
Supplement: Supplementary file 1 [file Table1.docx]

**Supplementary Table (ST) 1: Proinflammatory-related excluded articles.**

|  | **Author & Year** | **Title** | **Reason** |
| --- | --- | --- | --- |
| 1 | Al Deeb et al., 2020 | Clinical and immunological peri-implant parameters among cigarette and electronic smoking patients treated with photochemotherapy: A randomized controlled clinical trial | peri-implant |
| 2 | Almeda, et al., 2023 | E-cigarette vape promotes cell cycle progression and inflammation in 3D pre-clinical oral spheroid models | Poster Abstract |
| 3 | AlQahtani, et al., 2018 | Clinical peri-implant parameters and inflammatory cytokine profile among smokers of cigarette, e-cigarette, and waterpipe | peri-implant |
| 4 | ArRejaie, et al. 2019 | Proinflammatory cytokine levels and peri-implant parameters among cigarette smokers, individuals vaping electronic cigarettes, and non-smokers | peri-implant |
| 5 | Auschwitz, et al. 2023 | Mechanisms of E-Cigarette Vape-Induced Epithelial Cell Damage | Review |
| 6 | Baniulyte, et al., 2023 | Do e-cigarettes have a part to play in peri-implant diseases? | Commetart article |
| 7 | Javed, F., et al., 2019 | Tobacco-product usage as a risk factor for dental implants | Review |
| 8 | Mittal, A., et al., 2022 | Pathologic Alterations in Both the Microbiome and Immunomodulatory Phenotype of the Oropharynx in Healthy Electronic Cigarette Users | Conference Abstract |
| 9 | Mittal, et al., 2021 | Decreased Immunomodulatory and Inflammatory Cytokines in the Saliva of Healthy Electronic Cigarette Vapers | Conference Abstract |
| 10 | Mourão, et al., 2023 | What is the impact of e-cigarettes on periodontal stem cells as revealed by transcriptomic analyses? | Commetart article |
| 11 | Ralho, et al., 2019 | Effects of Electronic Cigarettes on Oral Cavity: A Systematic Review | Review |
| 12 | Sarles, et al., 2024 | Clinical Biomarkers to Evaluate Next Generation Inhaled Tobacco Products’ Effect in the Oral Cavity: a Narrative Review | Review |
| 13 | Tsai, et al., 2020 | Cell invasion, RAGE expression, and inflammation in oral squamous cell carcinoma (OSCC) cells exposed to e-cigarette flavoring | Data not related to Caries or proinflammatory |
| 14 | Youssef, et al., 2023 | The effect of electronic cigarette use on peri-implant conditions in men: a systematic review and meta-analysis | Review |
| 15 | Zięba, S., et al., 2024 | Can smoking alter salivary homeostasis? A systematic review on the effects of traditional and electronic cigarettes on qualitative and quantitative saliva parameters | Review |
| 16 | AlQahtani, et al., 2022 | Levels of whole salivary advanced glycation end products and interleukin‑17 and peri‑implant clinical and radiographic status in patients with osteoporosis at 6‑years’ follow‑up | peri-implant |
| 17 | AlHamoudi et al., 2020 | Effect of scaling and root planing on the expression of anti-inflammatory cytokines (IL-4, IL-9, IL-10, and IL-13) in the gingival crevicular fluid of electronic cigarette users and non-smokers with moderate chronic periodontitis | Data for Anti-inflammatory markers |
| 18 | Aljaser et al, 2021 | The effect of conventional versus electronic cigarette use on treatment outcomes of peri‑implant disease | peri-implant |

**Supplementary Table (ST) 2: Caries-related excluded articles.**

|  | **Author & Year** | **Title** | **Reason** |
| --- | --- | --- | --- |
| 1 | Abdelmonem, et al., 2019 | Effect of vaping on the oral cavity | Meeting abstract |
| 2 | Afrashtehfar, et al., 2021 | Vape labelling fails to report possible dental erosion and caries induced by e-cigarette fluids | Commetart article |
| 3 | Amaral, et al., 2023 | Electronic nicotine delivery systems (ENDS): a strategy for smoking cessation or a new risk factor for oral health? | Commetart article |
| 4 | Amaral, et al., 2023 | Is there evidence that e-cigarettes promote an increased risk of dental caries? | Commetart article |
| 5 | Cameron, al., 2024 | Current thinking about the effects of e-cigarettes on oral cancer risk | Review |
| 6 | Coll, et al., 2023 | Is there a significant difference in the oral microbiome in vapers vs non-vapers? | Commetart article |
| 7 | Esteban-Lopez, et al., 2022 | Health effects and known pathology associated with the use of E-cigarettes | Review |
| 8 | Hasan, et al.,2022 | Electronic Cigarette Vapour and the Impacts on Oral Health: A Review | Review |
| 9 | Irusa, et al., 2020 | Potential oral health effects of e-cigarettes and vaping: A review and case reports | Review |
| 10 | Ismail, et al., 2019 | Effect of electronic cigarette usage on oral health: A 6-month prospective study | Overlaped data |
| 11 | Javed, et al. 2019 | Tobacco-product usage as a risk factor for dental implants | Review |
| 12 | Jeong, et al., 2020 | Associations of electronic and conventional cigarette use with periodontal disease in South Korean adults | Data not related to Caries or proinflammatory |
| 13 | Majid, et al., 2024 | Salivary lipid changes in young adult tobacco smokers and e-cigarette users: a hidden risk to oral health? | Commetart article |
| 14 | Ralho, et al. 2019 | Effects of Electronic Cigarettes on Oral Cavity: A Systematic Review | Review |
| 15 | Sarles, et al. 2024 | Clinical Biomarkers to Evaluate Next Generation Inhaled Tobacco Products’ Effect in the Oral Cavity: a Narrative Review | Review |
| 16 | Sultan, et al., 2021 | Electronic nicotine delivery systems: Oral health implications and oral cancer risk | Review |
| 17 | Umerji, et al. 2018 | Cariogenicity of e-cigarettes | Review |
| 18 | Yang, et al. 2023 | Oral microbiome of electronic cigarette users: A cross-sectional exploration | Data not related to Caries or proinflammatory |
| 19 | Yeung, et al. 2023 | Is the use of tobacco products, especially electronic nicotine delivery systems (ENDS), associated with the incidence of oral health outcomes among US adults? | Commetart article |
| 20 | Dalrymple, et al., 2021 | Enamel staining with e-cigarettes, tobacco heating products and modern oral nicotine products compared with cigarettes and snus: An in vitro study | Data not related to Caries or proinflammatory |
| 21 | Caldwell, et al., 2020 | E-cigarette vapor exposure skews competition between colonizing oral Streptococci to allow biofilm formation of S. mutans and activation of stress and survival pathways in the oral cavity | Poster Abstract |

**Supplementary Table (ST) 3: PRISMA Checklist for the systematic review:**

| **Section and Topic** | **Item #** | **Checklist item** | **Location where item is reported** |
| --- | --- | --- | --- |
| **TITLE** | | |  |
| Title | 1 | Identify the report as a systematic review. | P1 |
| **ABSTRACT** | | |  |
| Abstract | 2 | See the PRISMA 2020 for Abstracts checklist. | P1 |
| **INTRODUCTION** | | |  |
| Rationale | 3 | Describe the rationale for the review in the context of existing knowledge. | P2 |
| Objectives | 4 | Provide an explicit statement of the objective(s) or question(s) the review addresses. | P2 |
| **METHODS** | | |  |
| Eligibility criteria | 5 | Specify the inclusion and exclusion criteria for the review and how studies were grouped for the syntheses. | P3 |
| Information sources | 6 | Specify all databases, registers, websites, organisations, reference lists and other sources searched or consulted to identify studies. Specify the date when each source was last searched or consulted. | P3 |
| Search strategy | 7 | Present the full search strategies for all databases, registers and websites, including any filters and limits used. | P3 |
| Selection process | 8 | Specify the methods used to decide whether a study met the inclusion criteria of the review, including how many reviewers screened each record and each report retrieved, whether they worked independently, and if applicable, details of automation tools used in the process. | P3 |
| Data collection process | 9 | Specify the methods used to collect data from reports, including how many reviewers collected data from each report, whether they worked independently, any processes for obtaining or confirming data from study investigators, and if applicable, details of automation tools used in the process. | P3,4 |
| Data items | 10a | List and define all outcomes for which data were sought. Specify whether all results that were compatible with each outcome domain in each study were sought (e.g. for all measures, time points, analyses), and if not, the methods used to decide which results to collect. | P4 |
|  | 10b | List and define all other variables for which data were sought (e.g. participant and intervention characteristics, funding sources). Describe any assumptions made about any missing or unclear information. | P4 |
| Study risk of bias assessment | 11 | Specify the methods used to assess risk of bias in the included studies, including details of the tool(s) used, how many reviewers assessed each study and whether they worked independently, and if applicable, details of automation tools used in the process. | P4 |
| Effect measures | 12 | Specify for each outcome the effect measure(s) (e.g. risk ratio, mean difference) used in the synthesis or presentation of results. | P6 |
| Synthesis methods | 13a | Describe the processes used to decide which studies were eligible for each synthesis (e.g. tabulating the study intervention characteristics and comparing against the planned groups for each synthesis (item #5)). | P5 |
|  | 13b | Describe any methods required to prepare the data for presentation or synthesis, such as handling of missing summary statistics, or data conversions. |  |
|  | 13c | Describe any methods used to tabulate or visually display results of individual studies and syntheses. |  |
|  | 13d | Describe any methods used to synthesize results and provide a rationale for the choice(s). If meta-analysis was performed, describe the model(s), method(s) to identify the presence and extent of statistical heterogeneity, and software package(s) used. |  |
|  | 13e | Describe any methods used to explore possible causes of heterogeneity among study results (e.g. subgroup analysis, meta-regression). |  |
|  | 13f | Describe any sensitivity analyses conducted to assess robustness of the synthesized results. |  |
| Reporting bias assessment | 14 | Describe any methods used to assess risk of bias due to missing results in a synthesis (arising from reporting biases). | Fig1 |
| Certainty assessment | 15 | Describe any methods used to assess certainty (or confidence) in the body of evidence for an outcome. | P4 |
| **RESULTS** | | |  |
| Study selection | 16a | Describe the results of the search and selection process, from the number of records identified in the search to the number of studies included in the review, ideally using a flow diagram. | P5, Fig 2 |
|  | 16b | Cite studies that might appear to meet the inclusion criteria, but which were excluded, and explain why they were excluded. | P5,6 Fig 2, supl file |
| Study characteristics | 17 | Cite each included study and present its characteristics. | P 6,7, T 1-3 |
| Risk of bias in studies | 18 | Present assessments of risk of bias for each included study. | Fig 1 |
| Results of individual studies | 19 | For all outcomes, present, for each study: (a) summary statistics for each group (where appropriate) and (b) an effect estimate and its precision (e.g. confidence/credible interval), ideally using structured tables or plots. | T 1-3 |
| Results of syntheses | 20a | For each synthesis, briefly summarise the characteristics and risk of bias among contributing studies. | P7 |
|  | 20b | Present results of all statistical syntheses conducted. If meta-analysis was done, present for each the summary estimate and its precision (e.g. confidence/credible interval) and measures of statistical heterogeneity. If comparing groups, describe the direction of the effect. | P7, Fig 3 |
|  | 20c | Present results of all investigations of possible causes of heterogeneity among study results. | P8 |
|  | 20d | Present results of all sensitivity analyses conducted to assess the robustness of the synthesized results. | P8 |
| Reporting biases | 21 | Present assessments of risk of bias due to missing results (arising from reporting biases) for each synthesis assessed. | P6, Fig 1 |
| Certainty of evidence | 22 | Present assessments of certainty (or confidence) in the body of evidence for each outcome assessed. | P7 |
| **DISCUSSION** | | |  |
| Discussion | 23a | Provide a general interpretation of the results in the context of other evidence. | P8 |
|  | 23b | Discuss any limitations of the evidence included in the review. | P9 |
|  | 23c | Discuss any limitations of the review processes used. | P9 |
|  | 23d | Discuss implications of the results for practice, policy, and future research. | P9 |
| **OTHER INFORMATION** | | |  |
| Registration and protocol | 24a | Provide registration information for the review, including register name and registration number, or state that the review was not registered. | P3 |
|  | 24b | Indicate where the review protocol can be accessed, or state that a protocol was not prepared. | P3 |
|  | 24c | Describe and explain any amendments to information provided at registration or in the protocol. | P3 |
| Support | 25 | Describe sources of financial or non-financial support for the review, and the role of the funders or sponsors in the review. | P10 |
| Competing interests | 26 | Declare any competing interests of review authors. | P10 |
| Availability of data, code and other materials | 27 | Report which of the following are publicly available and where they can be found: template data collection forms; data extracted from included studies; data used for all analyses; analytic code; any other materials used in the review. | P10 |
